# Effects of Light Spectra on Nutritional Composition in Juvenile *Sinonovacula constricta* (Lamarck 1818) and Transcriptomic Analysis

**DOI:** 10.1155/2024/5575475

**Published:** 2024-07-29

**Authors:** Mengqi Zhang, Fei Kong, Deshui Chen, Xiaojun Yan, Zhaoshou Ran, Jilin Xu

**Affiliations:** ^1^Key Laboratory of Aquacultural Biotechnology Ministry of Education, Ningbo University, Ningbo 315211, Zhejiang, China; ^2^Fujian Dalai Seedling Technology Co. Ltd., Luoyuan 350600, Fujian, China; ^3^Key Laboratory of Marine Biotechnology of Zhejiang Province, Ningbo 315211, Zhejiang, China

## Abstract

The razor clam *Sinonovacula constricta*, a commercially important and nutritionally valuable bivalve species, has been found to display notable responses to different light spectra. While previous research has highlighted the influence of light spectra on the growth, feeding rate, and various physiological characteristics of *S. constricta*, its impact on the biochemical composition of this species remains unclear. Herein, we investigated the proximate, fatty acid, and amino acid compositions of *S. constricta* cultured under various light spectra, including white, violet, blue, cyan, green, yellow, red, and darkness. Furthermore, we explored the potential molecular mechanisms underlying these observations through transcriptomic analysis. The results indicate that the light spectrum has a significant impact on the growth, biochemical composition, and gene expression of juvenile *S. constricta*. Specifically, culturing *S. constricta* under the yellow light led to improved growth rate (1.09 ± 0.03%/day), higher levels of carbohydrate (26.27% ± 0.49%), crude lipid (11.99% ± 0.23%), energy contents (14,611.34 ± 1,067.01 kJ/kg), and essential amino acids (15.22 ± 0.01 g/kg), as well as increased proportions of polyunsaturated fatty acids (12.38 ± 0.31 *µ*g/mg). These findings suggest that yellow light may play a crucial role in enhancing the nutritional quality of *S. constricta*. Moreover, the transcriptomic analysis revealed that the yellow light treatment upregulated pathways related to fatty acid biosynthesis, glycine, serine, and threonine metabolism and fatty acid metabolism. This indicates that yellow light may influence nutrient metabolism regulation in *S. constricta*, potentially leading to the observed changes in biochemical composition. Overall, our study recommends cultivating juvenile *S. constricta* under yellow light to optimize their growth and nutritional value. Further research could delve deeper into the molecular mechanisms underlying the effects of different light spectra on *S. constricta* to enhance our understanding of how light influences aquaculture practices and the nutritional quality of seafood products.

## 1. Introduction

Light is a crucial environmental factor that profoundly regulates the growth, development, physiological processes, and biochemical composition of both terrestrial and aquatic animals [1, 2]. However, the composition of light in aquatic environments differs significantly from terrestrial conditions. As one descends into the sea, longer wavelengths in the red–yellow spectrum become rapidly attenuated, dominating shallow waters [3], while shorter wavelengths in the blue–green spectrum prevail in the deep sea [4, 5]. This variation in light composition has profound implications for aquatic life, especially for those living in intertidal environments [6, 7]. For example, certain shellfish, such as abalone (*Haliotis discu hannai*), reared under the red or orange light exhibited the greater survival rate, specific growth rate, and food intake, as well as the conversion efficiency of food compared to those in groups exposed to blue or green light [8].

The nutritional composition of aquatic animals plays a crucial role in ensuring human health, encompassing carbohydrates, lipids, proteins, polyunsaturated fatty acids (PUFA), and amino acids [9]. However, there has been limited research on the effects of light spectra on the nutritional composition of teleost fish. For example, studies have shown that tilapia exhibits the highest content of lipid under red light while displaying the lowest lipid content under blue light, coinciding with the higher protein levels [10]. Surprisingly, the effects of light spectra on nutritional compositions of bivalve mollusks remains largely unexplored and requires urgent attention.

The razor clam *Sinonovacula constricta* (Lamarck 1818) is a crucial species in the global aquaculture industry, primarily found in shallow subtidal areas along the coasts of China, Japan, and Korea [11]. In order to meet the increasing market demand and enhance the productivity and quality of the razor clam, researchers have studied various environmental factors affecting their growth, including dietary microalgae [12], salinity [13], and pH [14]. Recently, our previous study revealed that the survival and growth of *S. constricta* juveniles (shell length, 1.55 ± 0.13 mm) were significantly higher under optimal natural sunlight intensity (380 ± 145 lx) compared to stronger or weaker sunlight intensities [15]. Furthermore, our lab observed that juvenile *S. constricta* showed improved growth under specific light wavelengths, particularly yellow light conditions [16]. Despite these findings, the impact of light spectra on the nutritional composition of *S. constricta* remains unclear, and further research is needed to understand the underlying mechanisms behind this phenomenon.

Therefore, in this study, we conducted a comprehensive analysis of the nutritional composition of proximates (moisture, carbohydrate, crude protein, and crude lipid), amino acid, and fatty acids in juvenile *S. constricta* cultured under various light conditions (dark environment, white, violet, blue, cyan, green, yellow, and red light). Additionally, leveraging RNA sequencing is a powerful tool for understanding functional genes and pathways in environment-related stresses to explore the underlying molecular mechanisms [17]. This study involved a transcriptomic analysis focusing on the selected representative light spectra, aiming to elucidate how *S. constricta* respond to diverse light spectra, thereby modulating its nutritional composition.

## 2. Materials and Methods

This study was carried out following the guidelines and with the approval of the Animal Research and Ethics Committees of Ningbo University.

### 2.1. Acclimation of Juvenile *S. constricta*

Healthy individuals of *S. constricta* (mean ± SD, shell length × width: 1.559 ± 0.071 cm × 0.547 ± 0.029 cm) were obtained from Fujian Dalai Seedling Technology Co., Ltd. (Ningde, Fujian Province, China). They were randomly sprinkled into 24 aquariums (dimensions: length × width × height, 20 × 20 × 20 cm) placed in a completely dark environment (photoperiod: 0-hr light/24-hr dark). The acclimation period lasted for 5 days. During acclimation, *S. constircta* were fed twice daily, at 8 : 00 AM and 6 : 00 PM, with fixed amounts of microalgae *Isochrysis galbana* and *Chaetoceros calcitrans* (1 : 1, v/v) at concentrations of ~450–600 cells/*μ*L, which was sufficient to cover the nutritional needs of shellfish at this stage. The culture temperature was maintained at 20 ± 1°C, and continuous aeration was provided. The seawater salinity was set at 25 psu (practical salinity units), which was processed through sedimentation and sand filtration. In preparation, fresh sea mud was dried at 200°C and filtered through a 100-*μ*m nylon sieve before being added to the aquariums.

### 2.2. Experimental Design

The LEDs used in this experiment were purchased from Shenzhen Yamingjie Intelligent Technology Co., Ltd. (Shenzhen, China), including white (peaking at 400–800 nm), violet (peaking at 397 nm), blue (peaking at 463 nm), cyan (peaking at 501 nm), green (peaking at 523 nm), yellow (peaking at 591 nm), red (peaking at 627 nm), and a dark setting. These LEDs were suspended at a height of 20 cm above the test tank. A spectroradiometer (PLA-30 Plant Lighting Analyzer, EVERFINE Photo-e-info Co., Ltd. Hangzhou, China) was employed to measure the spectral composition and light intensity. The photon flux density for each treatment group was set at 10.56 ± 0.10 *μ*mol/m^2^/s, in line with our prior findings [15]. Additionally, a 12-hr light/12-hr dark photoperiod (8 : 00 AM to 8 : 00 PM) was implemented.

The aquariums were lined with a 3-cm layer of sea mud. In each experimental tank, 30 juvenile *S. constricta* individuals were evenly distributed, and they were subjected to the eight light environments for a duration of 3 weeks. Each treatment was replicated three times to ensure reliability. Throughout the experiment, the feeding and other rearing conditions remained consistent with those during the acclimation, and half of the seawater in the tanks was replaced before each feeding to maintain water quality.

### 2.3. Growth Performance

At the end of the experiment, the razor clam was subjected to a 24 hr period of starvation to reduce the potential influence of microalgae on the results. Subsequently, all individuals in each treatment were measured to determine growth rates (shell length), survival rate, and specific growth rate (SGR). Muscle tissue was carefully dissected using sterilized surgical scissors and then stored at −80°C for further analysis. The survival rate and SGR for each treatment were calculated using the formula outlined in reference [18]:

(1) $$\begin{array}{c} \text{Survival rate }\left(\text{\% }\right)=\frac{\text{the number of surviving individuals at the end of the experiment}}{\text{the number of individuals at the begining of the experiment}}\times 100\%, \end{array}$$

(2) $$\begin{array}{c} \text{SGR }\left(\text{\%}/\text{day}\right)=\frac{\left(\text{the final shell length}-\text{the initial shell length}\right)\times 100\text{\% }}{\text{Rearing period }\left(\text{days}\right)}. \end{array}$$

### 2.4. Proximate Analysis

To determine the moisture content of the samples, they were subjected to oven (OHAUS MB45; Ohaus Corp., Melrose, MA, USA) drying at 105°C for 40 min until a constant weight was achieved. The crude protein content was quantified using an Automatic Kieldahl Apparatus (K9860, HANON, China). Specifically, 100 mg of lyophilized powder was combusted at 200°C for 10 min, 300°C for 10 min, and 400°C for 90 min using a graphite digestion apparatus SH220F (HANON, China). The resulting oxynitride was then reduced to nitrogen and quantified. For the determination of crude lipids, extraction was performed using a Soxhlet extractor following standard procedures, and the extracted lipid content was determined by weigh. Carbohydrate content was calculated using the Total Carbohydrate Content Kit (Geruisi, China). In this process, 100 mg of lyophilized powder was added to 750 *μ*L of the extraction solution and incubated at 95°C for 10 min. The absorbance was then measured at 500 nm to determine the carbohydrate content.

### 2.5. Energy Calculation

To confirm the effect of light spectra on the energy content of the body, the various energy reserve fractions (X: crude protein, carbohydrate, and crude lipid) were converted into energetic equivalents using their respective energy of combustion (E: 24 kJ/g protein, 17.5 kJ/g glycogen, and 39.5 kJ/g lipids) [19]. The energy content of *S. constricta* was calculated using the following equation:

(3) $$\begin{array}{c} E=\sum \left[\left(1-\text{moisture \%}\right)\times \text{body wet weight}\times \text{X \% }\times E\right]. \end{array}$$

### 2.6. Fatty Acid Analysis

The preparations of fatty acids were carried out following the methods described by Xu et al. [20]. In brief, 20 mg of lyophilized samples was first ground to fine powder using vitreous milling. The lipid was then extracted using 1 mL n-hexane and 1.5 mL of formyl chloride, with the addition of 15 *μ*L of C19 decanoic acid (1 mg/mL) as the internal standard. This mixture was heated at 60°C for 2 hr. Subsequently, 2.5 mL of 6% K_2_CO_3_ and 1 mL of n-hexane were added to the mixture. Finally, the supernatant liquid was transferred to an Eppendorf tube, centrifuged at 3,000 rpm/min for 10 min, filtered through a 0.22-*μ*m organic phase filtration membrane, and subjected to gas chromatography–mass spectrometry (GC-MS) analysis.

Fatty acid compositions were analyzed using a GC-MS analyzer (Agilent, 8890-5977B) equipped with a CD-2560 chromatographic column (100 m × 0.25 mm × 0.20 *μ*m, Anpel, China). Briefly, highly purified helium was supplied at a constant flow rate of 0.80 mL/min as the carrier gas, and the injector temperature was set at 250°C. A 1 *μ*L sample was injected with a split ratio of 1 : 5. Following injection, the oven temperature was initially held at 140°C for 5 min and then increased at a rate of 4°C/min to 240°C, where it was held for 20 min. The solvent cutoff time was set at 13.7 min. The mass spectrometer was operated with an electron impact ionization source at 70 eV. The ion source temperature and the quadrupole temperature were set at 230 and 150°C, respectively. The mass spectrometer scanned from m/z 40 to m/z 600.

The identifications of fatty acids were carried out using a combination of methods, including relative retention time, mass spectral databases (NIST14.L and Wiley7), and relevant published mass spectral data. The percentage composition of fatty acid was calculated as follows: (area of a specific fatty acid/area of total fatty acid) × 100%. The content composition of fatty acid was calculated using the following formula: (area of a specific fatty acid/area of the internal standard 19 : 0) × 30 *μ*g of 19 : 0/weight (mg) of lyophilized sample.

### 2.7. Amino Acid Analysis

The amino acid content was analyzed using an L-8900 automatic amino acid analyzer (Hitachi, Tokyo, Japan). Briefly, 0.1 g of lyophilized powder samples was hydrolyzed with 5 mL of HCl (6 mol/L) under a sand bath at 110°C for 24 hr. Following hydrolysis, the resulting hydrolysates were filtered through a membrane filter (0.25 *μ*m), and these filtered hydrolysates were then applied to the amino acid analyzer to determine amino acid contents (g/kg, dry weight).

### 2.8. Transcriptomic Analysis

Based on the growth results from previous experiments, two treatment groups of *S. constricta* were established: one in the dark and the other under yellow light, with each group triplicated. The experimental period lasted for 1 week, with all other experimental conditions remaining consistent with the aforementioned experimental design. At the end of the experiment, the entire tissue of the razor clam was collected, frozen with liquid nitrogen, and sent to Beijing Baimaike Biotechnology Co., Ltd. (China) for transcriptome sequencing.

After filtering out low-quality raw reads, including those containing only adaptor, sequences with more than 5% unknown nucleotides, or sequences with a Q20 score less than 20% (percentage of sequences with sequencing error rates <1%), the clean reads were aligned to the reference genome of *S. constricta* (Accession no. GCA_007844125.1) [21]. Differential gene expression between queen and worker honeybee were assessed using DESeq and *Q*-values. After that, gene abundance differences between those samples were calculated based on the ratio of the FPKM values. The false discovery rate (FDR) control method was used to identify the threshold of the *P* value in multiple tests to compute the significance of the differences. For subsequent analysis, only gene with an absolute value of the log2 ratio ≥ 2 and an FDR significance score <0.01 was considered. Once the mapped data were obtained, various analyses were conducted, including library quality assessment, structural level analysis, differential expression analysis, gene functional annotation, and functional enrichment analysis. Additionally, the Baimike cloud platform was utilized for in-depth data mining, which involved gene enrichment analysis, differential gene coexpression analysis, and other related analyses.

### 2.9. Statistical Analysis

The data were analyzed using one-way analysis of variance (ANOVA) in combination with multiple pairwise comparisons conducted by Duncan's multiple range test (SPSS 20.0) [22]. All values were expressed as mean ± SD, and a *P* < 0.05 was considered statistically significant.

To further investigate the effects of light spectra on fatty acid profiles in *S. constricta*, the fatty acids, considered as dependent data, underwent multivariate analyses. These analyses included principal compound analysis (PCA), partial least squares discriminant analysis (PLS-DA), and orthogonal partial least squares discrimination analysis (OPLS-DA). These analytical procedures were performed using the SIMCA software package V.17.0 [23].

## 3. Results

### 3.1. Growth Performance

**Figure 1:**
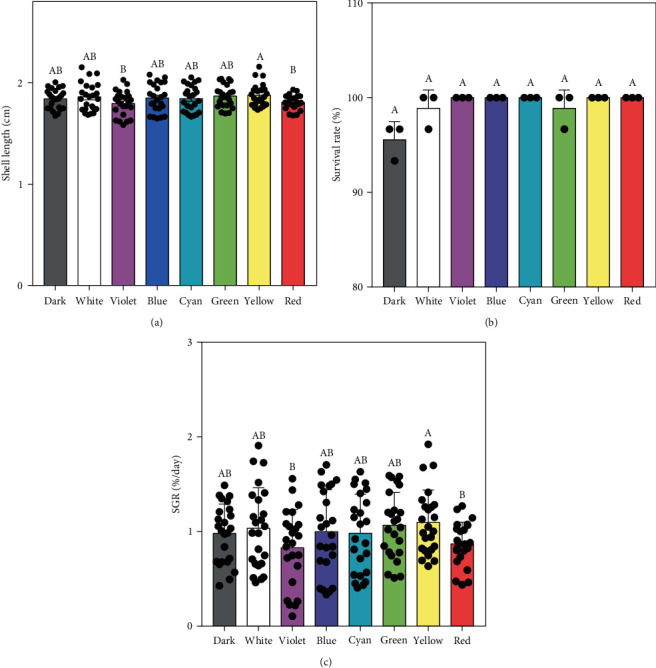
Growth performance of *S. constricta* cultured under different light spectra: (a) shell length (cm), (b) survival rate (%), and (c) SGR (%/day). Statistically significant differences are expressed with different letters (*P* < 0.05), while no differences are expressed using the same letter.

Figure 1 illustrates the significant impact of light spectra on the growth performance of juvenile *S. constricta*. Among the groups, the yellow light group exhibited the highest shell length (1.90 ± 0.11 cm) and SGR (1.09 ± 0.03%/day). These values were notably higher compared to those observed under red and violet lights. However, no significant differences were observed among the other light groups. Additionally, the survival rate of juvenile *S. constricta* exceeded 90% across all light groups, with no significant variations observed.

### 3.2. Moisture and Other Nutritional Composition

**Figure 2:**
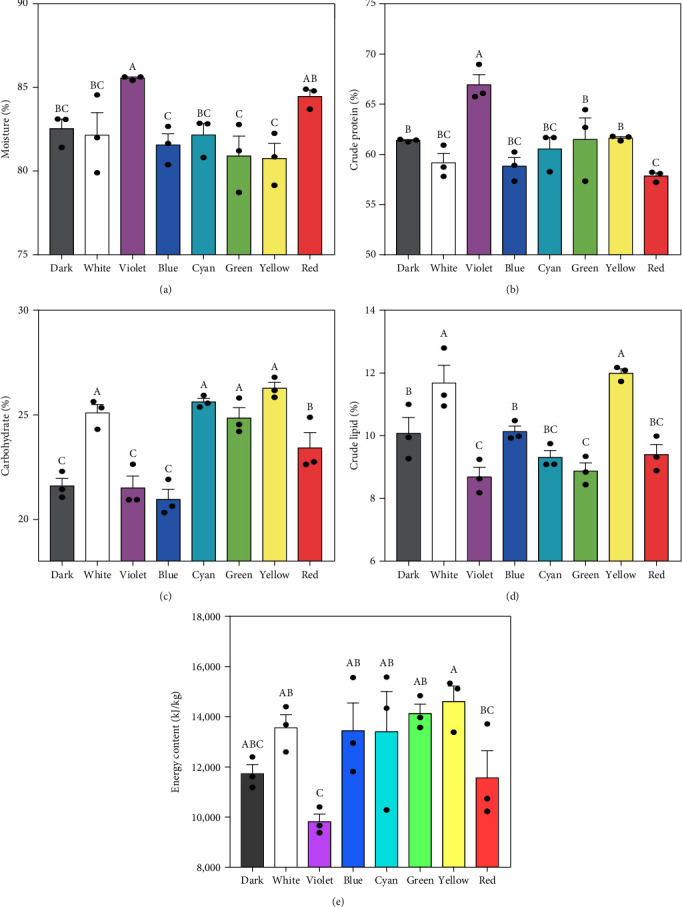
Proximate composition (%) of juvenile *S. constricta* cultured under different light spectra: (a) moisture (%, wet weight); (b) crude protein (%, dry weight); (c) carbohydrate (%, dry weight); (d) crude lipid (%, dry weight); and (e) energy content (kJ·kg^−1^, wet weight). Statistically significant differences are expressed with different letters (*P* < 0.05), while no differences are expressed using the same letter.

Figures 2(a), 2(b), 2(c), 2(d), and 2(e) display the moisture and other proximate compositions of juvenile *S. constricta* under different light spectra. Notably, the *S. constricta* subjected to the violet light exhibited the highest moisture content (85.56% ± 0.06%) and crude protein (66.93% ± 1.01%) while having the lowest crude lipid content (8.69% ± 0.53%) and energy content (9,814.12 ± 531.73 kJ·kg^−1^). Conversely, those under yellow light displayed the highest carbohydrate content (26.27% ± 0.49%), crude lipid (11.99% ± 0.23%), and energy content (14,611.34 ± 1,067.01 kJ·kg^−1^), along with the lowest moisture content (80.75% ± 0.89%). However, there were no significant differences observed between the blue light group and the other mentioned groups (yellow, green, cyan, white, and dark).

### 3.3. Fatty Acid Composition

**Figure 3:**
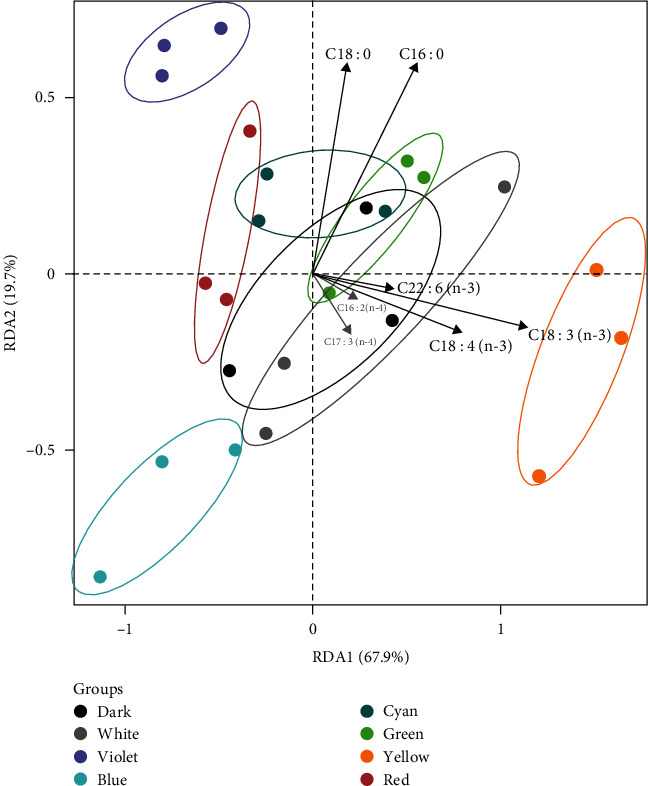
PCA score plots of fatty acid profile in juvenile *S. constricta* cultured under different light spectra. Each point represents the fatty acid profile of juveniles under the corresponding light spectrum. The horizontal and vertical coordinates represent the first two principal components, respectively, with the percentages in parentheses indicating the proportion of variables explained by each principal component.

Figure 3 presents the PCA results of the experiment, where each point on the plot corresponds to a sample cultured under a specific light condition. The PCA results revealed the significant differences in fatty acid composition among the yellow, violet, and blue light groups, while no notable differences were observed among the other light groups. Notably, higher levels of most PUFA with carbon chains of 18 and 22 were associated with the yellow light, whereas saturated fatty acids (SFA) with carbon chains of 16 and 18 showed a negative correlation with the blue light. To further investigate these principal components, the methods of PLS-DA and OPLS-DA were employed (Figures S1(a) and S1(b)). The results from these analyses further supported the assertion that the light spectrum plays a significant role in influencing the fatty acid composition of *S. constricta*.

Table 1 outlines the detailed results of fatty acid compositions in *S. constricta* cultured under various light spectra. Across the light treatment groups, 24 fatty acids were identified, except for the violet light (20 fatty acids) and the green light (23 fatty acids). The proportions of fatty acids mirrored their respective content ratios (Table S1). Remarkably, exposure to violet light promoted an increase of SFA while decreased the content and proportion of monounsaturated fatty acids (MUFA) and PUFA compared to the dark environment. In contrast, the yellow light exhibited the lowest content and proportion of C16 : 0 and C18 : 0, leading to the lowest SFA content and proportion. Additionally, yellow light resulted in the highest content and proportion of C16 : 2(n-4), *α*-linolenic acid (ALA), eicosapentaenoic acid (EPA), and docosahexaenoic acid (DHA), consequently showing the highest content and proportion of PUFA.

### 3.4. Amino Acid Composition

Tables 2 and S2 display the amino acid compositions in juvenile *S. constricta* cultured under various light spectra. It was observed that the content and proportion of TEAA were higher in the yellow light and dark environment, while the lowest levels were found in the green light group.

### 3.5. Transcriptomic Changes

RNA-seq analysis was conducted to investigate the influences of light spectra on juvenile *S. constricta* with a focus on the dark and yellow light groups due to their significant growth differences. The transcriptomic data from this study have been deposited in NCBI database under accession numbers SRR25678600-SRR25678605. As indicated in Table S3 and Figure S2, this strong correlation among the samples underscores their repeatability and stability, meeting the standards for further follow-up analysis.

**Figure 4:**
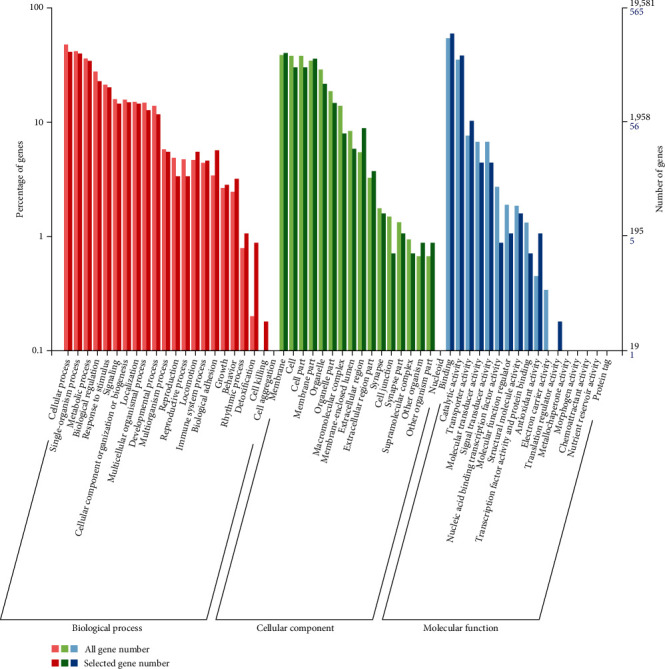
GO enrichment of all genes (light color) and DEGs (dark color) in juvenile *S. constricta* cultured under dark and yellow light. The horizontal axis denotes the GO terms, encompassing biological, cellular component, and molecular function categories. The left and right vertical axes represent the percentage and the number of genes classified in the corresponding term, respectively.

The results revealed that 57 gene ontology (GO) terms were regulated, as depicted in Figure 4. The identified GO terms predominantly centered around cellular process, single-organism process, and metabolic process in the biological process (BP). Regarding the molecular function (MF), the terms primarily revolved around membrane, cell, and cell part. Meanwhile, cellular component (CC) terms were mainly linked to binding, catalytic activity, and transporter activity.

**Figure 5:**
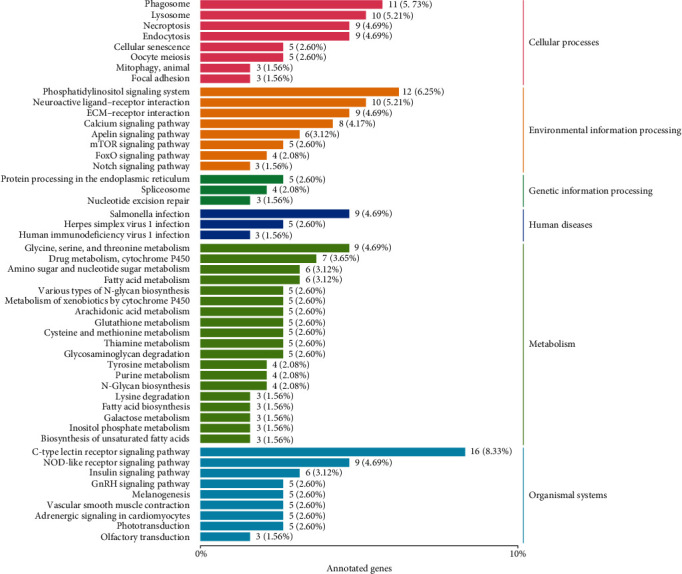
KEGG classification map of DEGs in juvenile *S. constricta* cultured under dark and yellow light. DEGs were categorized into six main groups: cellular process, environmental information processing, genetic information processing, human diseases, metabolism, and organismal systems.

Results obtained from the Kyoto Encyclopedia of Genes and Genomes (KEGG) analysis grouped the 1,005 GEGs into six pathways: cellular processes, environmental information processing, genetic information processing, human diseases, metabolism, and organismal systems (Figure 5). Further scrutiny of the DEGs revealed distinctions between the upregulated and downregulated genes (Table 3). The upregulated DEGs were significantly enriched in fatty acid biosynthesis; glycine, serine, and threonine metabolism; and amino sugar and nucleotide sugar metabolism, among others. Conversely, the downregulated DEGs were significantly enriched in 47 pathways, such as C-type lectin receptor signaling pathway, necroptosis, and NOD-like receptor signaling pathway.

## 4. Discussion

### 4.1. Growth Performance of Juvenile *S. constricta* Is Significantly Influenced by Light Spectra

The nutrient composition of shellfish is closely linked to microalgae [24]. Various light spectra under high light intensity can selectively promote the growth of different microalgae [25]. To minimize the influence of microalgae on the nutrient composition of razor clams, we fed them the same type of microalgae and used a moderate light intensity in this study.

This study demonstrated that the razor clam exhibited enhanced growth under yellow light. This finding aligns with Zhang et al. [16]'s observations, where significant improvements in the growth performance of juvenile *S. constricta* were noted under yellow light. Additionally, rainbow trout (*Oncorhynchus mykiss*) showed the highest growth performance when exposed to yellow light rather than blue and red light [26]. The preference for yellow light could be attributed to more efficient energy utilization, enabling a greater allocation of energy toward growth rather than expenditure [27]. These collective findings suggest that yellow light's promotion of aquatic animal growth might be a common phenomenon among species inhabiting shallow water areas, albeit with species-specific variations.

### 4.2. Proximate Composition of Juvenile *S. constricta* Varies Greatly under Different Light Spectra

Inappropriate light spectra prompt the razor clam to reorganize its metabolism, utilizing energy resources such as lipids, proteins, and carbohydrates to meet increased energy demands under light-induced stress. Our study observed that razor clams reared under yellow light exhibited higher lipid and carbohydrate contents, resulting in superior growth performance. Furthermore, our results showed that juvenile *S. constricta* exposed to yellow light had the lowest moisture content, potentially contributing to a more cohesive texture, as indicated previously [28]. Interestingly, energy content varied across light spectra. While dark settings are conventionally considered conducive for *S. constricta* growth due to their burrowing lifestyle, our findings challenged this notion. Contrary to expectations, darkness did not appear to be the ideal setting for the razor clam. Instead, specimens reared under yellow light contained a higher energy content, suggesting a favorable light environment for their growth.

### 4.3. Fatty Acid and Amino Acid Composition of Juvenile *S. constricta* are Regulated by Light Spectra

PCA, PLS-DA, and OPLS-DA are standard methods in multivariate statistical analysis. PCA evaluates data group repeatability and differences by retaining information contributing most to variance. Samples under different light spectra exhibited distinct distributions, highlighting significant differences in *S. constricta*'s fatty acid composition. Notably, substantial variations were observed in the razor clam's fatty acid composition between the yellow and violet light groups. However, further investigation is needed to determine whether this difference in fatty acids directly correlates with the observed growth variation in the razor clam.

*S. constricta* is the sole marine mollusk known to possess all necessary fatty acyl desaturases and elongases of very long-chain fatty acids required for LC-PUFA biosynthesis through the Sprecher pathway [29], suggesting its potential for endogenous biosynthesis of EPA and DHA—essential nutrients vital for bivalve early development [30]. Our investigation revealed that yellow light increased the proportion and content of PUFA, including EPA and DHA, while violet light had the opposite effect. Notably, other light conditions showed negligible variations compared to the dark environment. These findings strongly indicate that yellow light could potentially upregulate LC-PUFA biosynthesis in *S. constricta*.

Amino acids play a key role in food nutrition and various physiological functions [31]. Our investigation noted lower content of TEAA, TFAA, and TMAA under green light, while higher levels were observed under yellow light and in a dark environment. The underlying reasons for these variations warrant further investigation.

### 4.4. Transcriptome of Juvenile *S. constricta* Is Responsive to Light Spectra

Comparing the dark versus yellow light groups, KEGG pathway analysis highlighted enrichment primarily in fatty acid metabolism (Figure S7), amino acid metabolism (Figure S8), peroxisomes (Figure S9), and the cytochrome P450 (Figure S10) pathway. These pathways, known for their significance in nutritional metabolism, play essential roles in energy release and the synthesis of crucial biomolecules [32, 33]. Specifically, fatty acid metabolism is pivotal, serving as a primary energy source when oxidized in the presence of oxygen [34]. Amino acid metabolism contributes to the synthesis of body-specific proteins and peptides, potentially converting into carbohydrates, lipids, and certain nonessential amino acids [35]. Transcriptome analysis consistently revealed significant increases in the gene expressions of *fasn* and *acat* in the fatty acid metabolic pathway (Figure S3), and *gcdh*, *bhmt*, and *grhpr* in the amino acid metabolic pathway (Figure S4) in *S. constricta* under yellow light. Peroxisomes and cytochrome P450 pathways primarily facilitate cell oxidation–reduction and catalyzed reactions of bioactive molecules [36, 37]. Peroxisomes contain enzymes like catalase that deactivate toxins and participate in fatty acid oxidation, while cytochrome P450 enzymes are integral in synthesizing steroid hormones, metabolizing fat-soluble vitamins, and converting PUFA into bioactive molecules. Notably, transcriptome data indicated the upregulation of peroxisome and cytochrome P450 pathway genes under yellow light (Figures S5 and S6). Taken together, differences in FPKM values observed in the transcriptome indicate that yellow light induced the upregulation of genes associated with nutrient metabolism pathways, exerting a significant influence on the regulation of nutrient metabolism.

## 5. Conclusions

**Figure 6:**
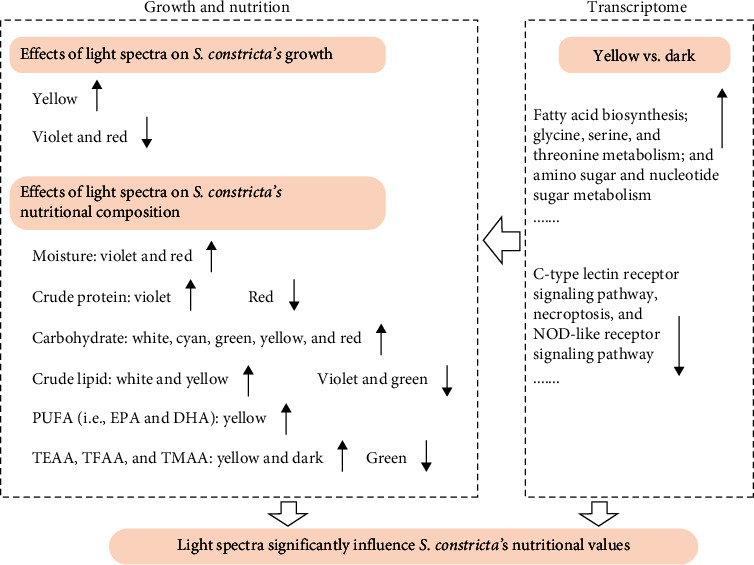
A brief overview of the results of the present study.

As summarized in Figure 6, our study demonstrated that yellow light significantly influenced the growth performance, lipid and carbohydrate content, PUFA levels (especially DHA), and amino acid content of juvenile *S. constricta*. These effects could be associated with the upregulation of specific DEGs enriched for GO terms and KEGG pathways. Notably, distinct gene expression patterns were observed between dark and yellow light conditions, particularly in metabolic pathways such as fatty acid biosynthesis; glycine, serine, and threonine metabolism; and fatty acid metabolism. Overall, cultivating juvenile *S. constricta* under yellow light conditions resulted in enhanced growth and nutritional values. Additionally, the transcriptomic insights provide a foundation for further research into the underlying mechanisms governing the influence of light spectra on this high-nutrition-value razor clam.

## Figures and Tables

**Table 1:** Fatty acid composition of juvenile *S. constricta* cultured under different light spectra.

Fatty acid	Content under different light spectra (*μ*g·mg^−1^)							
	Dark	White	Violet	Blue	Cyan	Green	Yellow	Red
C14 : 0	0.28 ± 0.03^b^	0.28 ± 0.03^b^	0.33 ± 0.04^b^	0.29 ± 0.02^b^	0.27 ± 0.08^b^	0.26 ± 0.00^b^	0.41 ± 0.01^a^	0.28 ± 0.02^b^
C16 : 0	4.81 ± 0.32^a^	4.61 ± 0.42^b^	4.83 ± 0.11^a^	3.93 ± 0.28^c^	4.88 ± 0.11^a^	4.74 ± 0.17^b^	4.93 ± 0.48^a^	4.45 ± 0.13^b^
C16 : 1 (n-7)	0.74 ± 0.05^bc^	0.97 ± 0.08^b^	0.70 ± 0.04^e^	0.83 ± 0.07^cd^	0.91 ± 0.06^bc^	0.76 ± 0.04^de^	1.15 ± 0.02^a^	0.76 ± 0.02^de^
C16 : 2 (n-4)	0.19 ± 0.01^d^	0.25 ± 0.01^c^	0.18 ± 0.01^d^	0.19 ± 0.01^d^	0.30 ± 0.03^b^	0.21 ± 0.01^cd^	0.63 ± 0.01^a^	0.20 ± 0.01^cd^
C17 : 0	0.05 ± 0.01^a^	0.05 ± 0.01^a^	n.d.	0.05 ± 0.01^a^	0.05 ± 0.00^a^	0.05 ± 0.00^a^	0.06 ± 0.01^a^	0.05 ± 0.00^a^
C17 : 3 (n-4)	0.26 ± 0.01^d^	0.36 ± 0.03^c^	n.d.	0.36 ± 0.04^c^	0.34 ± 0.02^c^	0.44 ± 0.01^b^	0.49 ± 0.01^a^	0.36 ± 0.01^c^
C18 : 0	3.06 ± 0.21^bc^	3.23 ± 0.37^bc^	3.63 ± 0.19^ab^	2.88 ± 0.13^c^	3.36 ± 0.05^abc^	3.83 ± 0.25^a^	3.35 ± 0.03^abc^	3.49 ± 0.44^ab^
C18 : 1 (n-9)	1.41 ± 0.11^b^	1.28 ± 0.11^b^	1.04 ± 0.03^c^	1.31 ± 0.10^b^	1.39 ± 0.12^b^	1.37 ± 0.04^b^	1.78 ± 0.05^a^	1.37 ± 0.02^b^
C18 : 1 (n-7)	1.37 ± 0.10^ab^	1.35 ± 0.12^ab^	1.16 ± 0.03^b^	1.16 ± 0.11^b^	1.42 ± 0.11^ab^	1.41 ± 0.01^ab^	1.72 ± 0.05^a^	1.37 ± 0.02^ab^
C18 : 2 (n-6)	0.13 ± 0.01^bc^	0.15 ± 0.01^ab^	0.12 ± 0.01^cd^	0.11 ± 0.01^d^	0.13 ± 0.01^c^	0.14 ± 0.00^bc^	0.16 ± 0.00^a^	0.13 ± 0.00^bc^
C18 : 3 (n-6)	0.24 ± 0.02^bc^	0.24 ± 0.02^bc^	n.d.	0.23 ± 0.01^c^	0.23 ± 0.01^c^	n.d.	0.30 ± 0.02^a^	0.27 ± 0.02^b^
C18 : 3 (n-3)	3.29 ± 0.28^bcd^	3.35 ± 0.44^bc^	2.62 ± 0.11^d^	2.78 ± 0.19^cd^	2.98 ± 0.22^bcd^	3.56 ± 0.15^ab^	4.21 ± 0.08^a^	2.89 ± 0.09^cd^
C18 : 4 (n-3)	2.26 ± 0.21^bc^	2.47 ± 0.26^b^	1.90 ± 0.08^d^	2.00 ± 0.12^cd^	2.22 ± 0.21^bc^	2.32 ± 0.11^b^	2.93 ± 0.07^a^	1.78 ± 0.06^cd^
C20 : 0	0.05 ± 0.00^a^	0.05 ± 0.01^a^	0.06 ± 0.00^a^	0.05 ± 0.01^a^	0.05 ± 0.00^a^	0.05 ± 0.00^a^	0.05 ± 0.00^a^	0.05 ± 0.00^a^
C20 : 1 (n-9)	0.13 ± 0.01^a^	0.14 ± 0.03^a^	0.11 ± 0.01^a^	0.11 ± 0.01^a^	0.13 ± 0.01^a^	0.13 ± 0.01^a^	0.14 ± 0.01^a^	0.12 ± 0.01^a^
C20 : 1 (n-7)	0.99 ± 0.07^de^	1.15 ± 0.11^bc^	0.91 ± 0.02^e^	0.91 ± 0.06^e^	1.08 ± 0.07^cd^	1.21 ± 0.04^b^	1.35 ± 0.09^a^	1.02 ± 0.01^de^
C20 : 2 (n-7)	0.09 ± 0.01^ab^	0.10 ± 0.01^a^	0.09 ± 0.00^ab^	0.09 ± 0.01^ab^	0.10 ± 0.01^a^	0.10 ± 0.00^a^	0.12 ± 0.00^a^	0.09 ± 0.00^ab^
20 : 4 (n-6)	0.12 ± 0.01^b^	0.11 ± 0.01^bc^	0.08 ± 0.00^d^	0.11 ± 0.01^bcd^	0.09 ± 0.01^cd^	0.10 ± 0.00^bcd^	0.16 ± 0.00^a^	0.15 ± 0.00^a^
20 : 3 (n-6)	0.05 ± 0.01^bc^	0.05 ± 0.01^b^	0.04 ± 0.00^cd^	0.03 ± 0.00^d^	0.05 ± 0.00^ab^	0.06 ± 0.00^a^	0.06 ± 0.00^a^	0.05 ± 0.00^bc^
20 : 5 (n-3)	0.26 ± 0.02^b^	0.25 ± 0.01^b^	0.17 ± 0.00^c^	0.20 ± 0.01^c^	0.26 ± 0.04^b^	0.24 ± 0.03^b^	0.32 ± 0.01^a^	0.24 ± 0.01^b^
22 : 2 (5,13)	0.13 ± 0.01^bc^	0.13 ± 0.01^b^	0.10 ± 0.00^d^	0.11 ± 0.01^cd^	0.13 ± 0.01^b^	0.14 ± 0.01^b^	0.17 ± 0.00^a^	0.12 ± 0.01^bc^
22 : 4 (n-6)	0.65 ± 0.04^bc^	0.69 ± 0.06^b^	0.50 ± 0.01^d^	0.58 ± 0.04^c^	0.65 ± 0.04^bc^	0.69 ± 0.04^b^	0.92 ± 0.03^a^	0.67 ± 0.02^b^
22 : 5 (n-6)	0.06 ± 0.01^b^	0.08 ± 0.01^a^	n.d.	0.05 ± 0.04^bc^	0.06 ± 0.00^b^	0.06 ± 0.00^b^	0.08 ± 0.00^a^	0.05 ± 0.00^bc^
22 : 6 (n-3)	1.30 ± 0.08^bc^	1.45 ± 0.12^b^	1.16 ± 0.03^c^	1.19 ± 0.08^c^	1.34 ± 0.10^bc^	1.48 ± 0.05^b^	1.77 ± 0.06^a^	1.32 ± 0.03^bc^
SFA	8.27 ± 0.51^bc^	8.24 ± 0.78^bc^	8.86 ± 0.17^a^	7.23 ± 0.46^c^	8.63 ± 0.24^b^	8.73 ± 0.41^b^	8.82 ± 0.47^a^	8.35 ± 0.45^bc^
MUFA	4.66 ± 0.36^bc^	4.90 ± 0.47^bc^	3.94 ± 0.12^c^	4.35 ± 0.34^b^	4.94 ± 0.36^b^	4.90 ± 0.12^b^	6.17 ± 0.22^a^	4.62 ± 0.08^bc^
PUFA	9.08 ± 0.72^bc^	9.75 ± 1.07^b^	6.83 ± 0.26^d^	8.10 ± 0.55^cd^	8.94 ± 0.74^bc^	9.59 ± 0.38^b^	12.38 ± 0.31^a^	8.38 ± 0.09^c^
TFA	22.02 ± 1.52^bc^	22.90 ± 1.53^b^	19.64 ± 0.53^d^	19.68 ± 1.33^cd^	22.52 ± 1.33^b^	23.24 ± 0.92^b^	27.39 ± 0.88^a^	21.37 ± 0.52^bcd^

*Note*. SFA, saturated fatty acids; MUFA, monounsaturated fatty acids; PUFA, polyunsaturated fatty acids; TFA, total fatty acids; n.d., not detected. Date is expressed as mean ± SD (*n* = 3). Values in the same row sharing the same letter were not significantly different (*P* < 0.05).

**Table 2:** Amino acid composition of juvenile *S. constricta* cultured under different light spectra.

Amino acid	Content under different light spectra (×10 g (kg))							
	Dark	White	Violet	Blue	Cyan	Green	Yellow	Red
Thr^1^	1.95 ± 0.01^a^	1.85 ± 0.07^ab^	1.89 ± 0.02^ab^	1.85 ± 0.04^ab^	1.78 ± 0.08^bc^	1.71 ± 0.02^c^	1.93 ± 0.00^a^	1.82 ± 0.04^abc^
Val^1^	1.85 ± 0.01^a^	1.78 ± 0.05^bc^	1.84 ± 0.00^ab^	1.80 ± 0.04^ab^	1.72 ± 0.03^cd^	1.70 ± 0.04^d^	1.86 ± 0.01^a^	1.77 ± 0.05^bc^
Ile^1^	1.95 ± 0.01^a^	1.87 ± 0.05^bcd^	1.94 ± 0.00^ab^	1.88 ± 0.02^abc^	1.80 ± 0.03^de^	1.79 ± 0.05^e^	1.94 ± 0.00^a^	1.86 ± 0.06^cde^
Leu^1,3^	3.97 ± 0.02^a^	3.88 ± 0.10^ab^	3.97 ± 0.02^a^	3.85 ± 0.01^b^	3.73 ± 0.01^cd^	3.68 ± 0.04^d^	3.98 ± 0.01^a^	3.80 ± 0.13^bc^
Met^1,3^	1.03 ± 0.00^a^	1.01 ± 0.01^a^	1.01 ± 0.00^a^	1.02 ± 0.02^a^	0.95 ± 0.04^bc^	0.91 ± 0.04^c^	1.04 ± 0.01^a^	1.00 ± 0.03^ab^
Phe^1,3^	1.34 ± 0.00^a^	1.30 ± 0.03^abc^	1.33 ± 0.00^ab^	1.30 ± 0.04^abc^	1.28 ± 0.04^cd^	1.24 ± 0.02^d^	1.34 ± 0.00^a^	1.28 ± 0.03^bcd^
Lys^1,3^	3.10 ± 0.01^a^	3.05 ± 0.08^ab^	3.11 ± 0.01^a^	3.01 ± 0.03^ab^	2.98 ± 0.06^bc^	2.90 ± 0.03^c^	3.11 ± 0.00^a^	2.98 ± 0.08^bc^
Ser^2^	1.93 ± 0.02^a^	1.93 ± 0.04^a^	1.95 ± 0.00^a^	1.79 ± 0.05^b^	1.81 ± 0.09^b^	1.75 ± 0.00^b^	1.94 ± 0.01^a^	1.81 ± 0.05^b^
Pro^2^	0.47 ± 0.00^a^	0.47 ± 0.01^a^	0.47 ± 0.00^a^	0.46 ± 0.01^ab^	0.46 ± 0.02^ab^	0.44 ± 0.00^b^	0.48 ± 0.00^a^	0.46 ± 0.01^a^
Ala^2^	7.40 ± 0.05^a^	7.41 ± 0.02^a^	7.32 ± 0.51^a^	7.17 ± 0.13^a^	6.91 ± 0.45^ab^	6.66 ± 0.11^b^	6.91 ± 0.10^ab^	7.07 ± 0.20^ab^
Glu^2,3^	5.75 ± 0.01^a^	5.64 ± 0.15^ab^	5.78 ± 0.04^a^	5.63 ± 0.07^ab^	5.42 ± 0.02^c^	5.36 ± 0.05^c^	5.77 ± 0.05^a^	5.46 ± 0.24^bc^
Gly^2,3^	2.28 ± 0.01^a^	2.14 ± 0.09^a^	2.31 ± 0.22^a^	2.20 ± 0.16^a^	2.17 ± 0.33^a^	2.32 ± 0.00^a^	2.48 ± 0.05^a^	2.16 ± 0.24^a^
Asp^2,3^	3.94 ± 0.00^a^	3.83 ± 0.09^ab^	3.92 ± 0.02^a^	3.83 ± 0.07^ab^	3.65 ± 0.08^cd^	3.62 ± 0.03^d^	3.95 ± 0.00^a^	3.76 ± 0.12^bc^
Arg^3^	3.13 ± 0.00^ab^	3.14 ± 0.17^ab^	3.10 ± 0.06^ab^	3.18 ± 0.17^ab^	3.15 ± 0.22^ab^	2.99 ± 0.06^b^	3.31 ± 0.14^a^	3.11 ± 0.08^ab^
Tyr^3^	1.15 ± 0.01^ab^	1.13 ± 0.04^ab^	1.13 ± 0.01^ab^	1.14 ± 0.04^ab^	1.14 ± 0.09^ab^	1.06 ± 0.02^b^	1.17 ± 0.02^a^	1.11 ± 0.05^ab^
Cys	0.29 ± 0.00^a^	0.29 ± 0.01^a^	0.31 ± 0.00^a^	0.32 ± 0.03^a^	0.30 ± 0.03^a^	0.25 ± 0.01^b^	0.32 ± 0.01^a^	0.30 ± 0.00^a^
His	0.85 ± 0.00^ab^	0.83 ± 0.02^ab^	0.85 ± 0.00^ab^	0.83 ± 0.02^ab^	0.79 ± 0.01^c^	0.79 ± 0.00^c^	0.85 ± 0.00^a^	0.82 ± 0.01^b^
TEAA	15.23 ± 0.09^a^	14.77 ± 0.40^bc^	15.13 ± 0.07^ab^	14.74 ± 0.14^bc^	14.28 ± 0.10^de^	13.97 ± 0.25^e^	15.22 ± 0.01^a^	14.54 ± 0.42^cd^
TFAA	21.79 ± 0.06^a^	21.44 ± 0.23^ab^	21.77 ± 0.21^a^	21.12 ± 0.38^bc^	20.44 ± 0.32^de^	20.17 ± 0.03^e^	21.55 ± 0.01^ab^	20.75 ± 0.63^cd^
TMAA	25.73 ± 0.10^ab^	25.16 ± 0.67^abc^	25.70 ± 0.42^ab^	25.21 ± 0.51^abc^	24.50 ± 0.65^cd^	24.12 ± 0.31^d^	26.18 ± 0.05^a^	24.71 ± 0.96^cd^
TAA	42.46 ± 0.17^a^	41.61 ± 0.87^ab^	42.31 ± 0.04^a^	41.35 ± 0.71^ab^	40.12 ± 0.12^cd^	39.25 ± 0.31^d^	42.45 ± 0.18^a^	40.67 ± 1.18^bc^

*Note*. ^1^Essentital amino acids. ^2^Flavor amino acids. ^3^Medicinal amino acids. Thr, threonine; Val, valine; Ile, isoleucine; Leu, leucine; Met, methionine; Phe, phenylalanine; Lys, lysine; Ser, serine; Pro, proline; Ala, alanine; Glu, glutamic acid; Gly, glycine; Asp, aspartic acid; Arg, arginine; Tyr, tyrosine; Cys, cysteine; His, histidine; TEAA, total essential amino acids; TFAA, total flavor amino acids; TMAA, total medicinal amino acids; and TAA, total amino acids. Date is expressed as mean ± SD (*n* = 3). Values in the same row sharing the same letter were not significantly different (*P* < 0.05).

**Table 3:** The increased and decreased KEGG pathways significantly enriched by the DEGs in juvenile *S. constricta* cultured under yellow light compared to dark conditions (*P* < 0.05).

Term	ID	Input number	Background number	*P* value
Increased KEGG pathways				
Fatty acid biosynthesis	ko00061	3	37	0.013278
Glycine, serine, and threonine metabolism	ko00260	5	109	0.015622
Amino sugar and nucleotide sugar metabolism	ko00520	5	125	0.026541
Drug metabolism, cytochrome P450	ko00982	3	52	0.034326
Glycosaminoglycan degradation	ko00531	3	56	0.039470
Fatty acid metabolism	ko01212	4	96	0.040349
Steroid hormone biosynthesis	ko00140	2	25	0.044215
Thiamine metabolism	ko00730	2	25	0.044215
Decreased KEGG pathways				
C-type lectin receptor signaling pathway	ko04625	12	140	5.02E-07
Necroptosis	ko04217	7	57	1.31E-05
Salmonella infection	ko05132	7	72	6.14E-05
NOD-like receptor signaling pathway	ko04621	7	75	8.00E-05
Calcium signaling pathway	ko04020	7	90	0.000253
Phosphatidylinositol signaling system	ko04070	10	206	0.000596
Apelin signaling pathway	ko04371	6	76	0.000657
Melanogenesis	ko04916	5	58	0.001264
Phototransduction	ko04744	5	60	0.001474
GnRH signaling pathway	ko04912	5	66	0.002257

## Data Availability

Data for this research article were available from the corresponding authors by reasonable request.

## Abbreviations

LED: Light-emitting diode
FA: Fatty acids
SFA: Saturated fatty acids
MUFA: Monounsaturated fatty acids
PUFA: Polyunsaturated fatty acids
LC-PUFA: Long-chain polyunsaturated fatty acids
ALA: *α*-Linolenic acid
EPA: Eicosapentaenoic acid
DHA: Docosahexaenoic acid
TFAA: Total flavor amino acids
TEAA: Total essential amino acids
TMAA: Total medicinal amino acids
TAA: Total amino acids
Thr: Threonine
Val: Valine
Ile: Isoleucine
Leu: Leucine
Met: Methionine
Phe: Phenylalanine
Lys: Lysine
Ser: Serine
Pro: Proline
Ala: Alanine
Glu: Glutamic acid
Gly: Glycine
Asp: Aspartic acid
Arg: Arginine
Tyr: Tyrosine
Cys: Cysteine
His: Histidine
GO: Gene Ontology
BP: Biological process
MF: Molecular function
CC: Cellular component
DEGs: Differentially expressed genes
KEGG: Kyoto Encyclopedia of Genes and Genomes.

## Additional Points

*Highlights*. Nutrients of *S. constricta* are significantly impacted by culturing light spectra. Yellow light significantly improved the growth rate of *S. constricta*. Yellow light significantly enhanced the nutritional quality of *S. constricta*. Yellow light may be beneficial for the nutritional metabolism of *S. constricta*.

## Supplementary Materials

Supplementary Materials The supplementary of this manuscript describes the specific page number of each part of the manuscript, including the purpose, methods, results, and other content of the experiment.
