# Supplementary material for: Effects of Light Spectra on Nutritional Composition in Juvenile Sinonovacula constricta (Lamarck 1818) and Transcriptomic Analysis
Source: Aquac Nutr. 2024 Jul 29;2024:5575475. doi: 10.1155/2024/5575475 (PMC11458319; doi:10.1155/2024/5575475)
Supplement: Supplementary Materials — The supplementary of this manuscript describes the specific page number of each part of the manuscript, including the purpose, methods, results, and other content of the experiment. [file 5575475.f1.docx]

**Supplementary Figures**


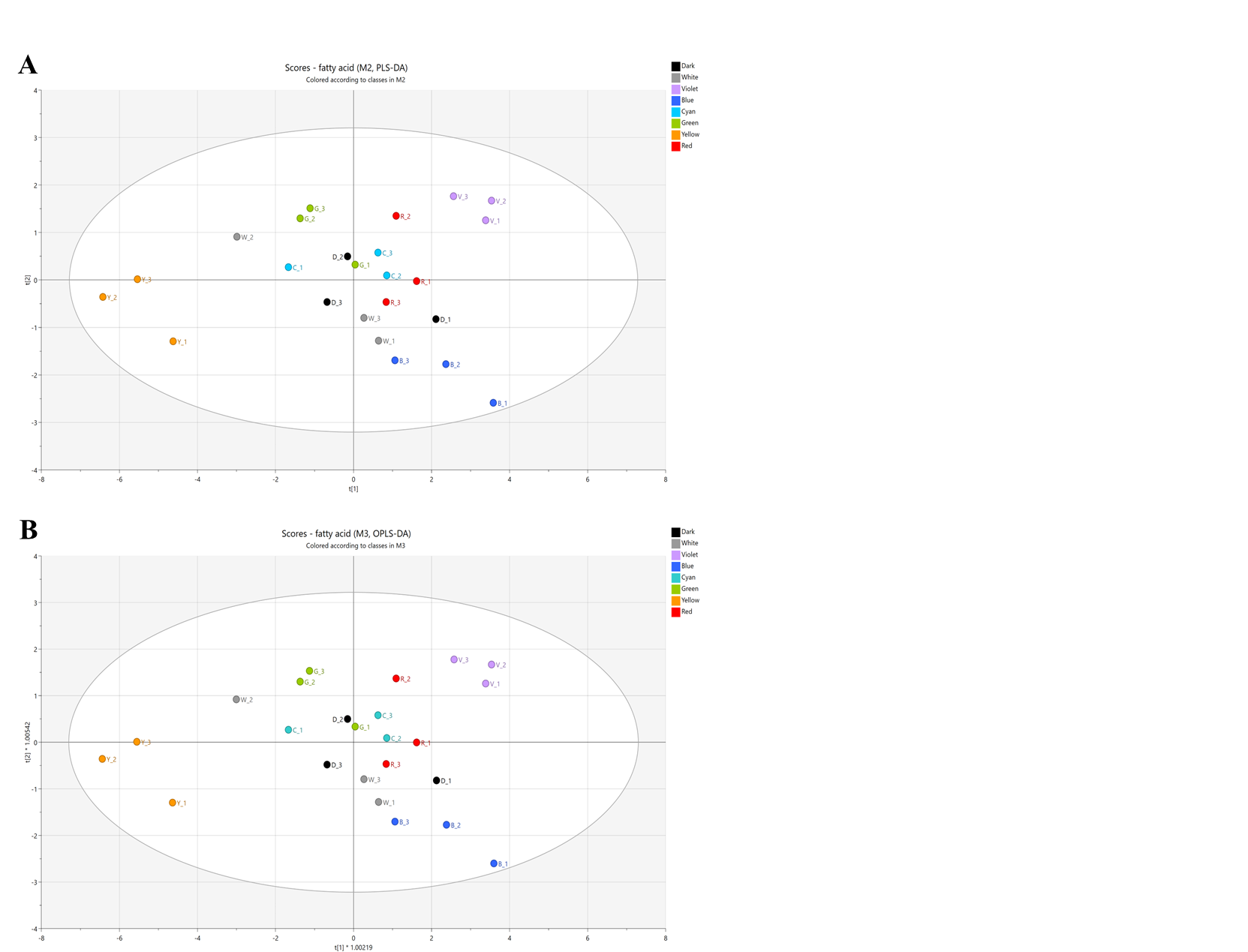


**Figure S1.** PLS-DA (A) and DPLS-DA (B) scores plots of fatty acid compositions in *S. constricta* cultured under different light spectra. Each point represents the FA profile of juveniles cultured under corresponding light spectrum.


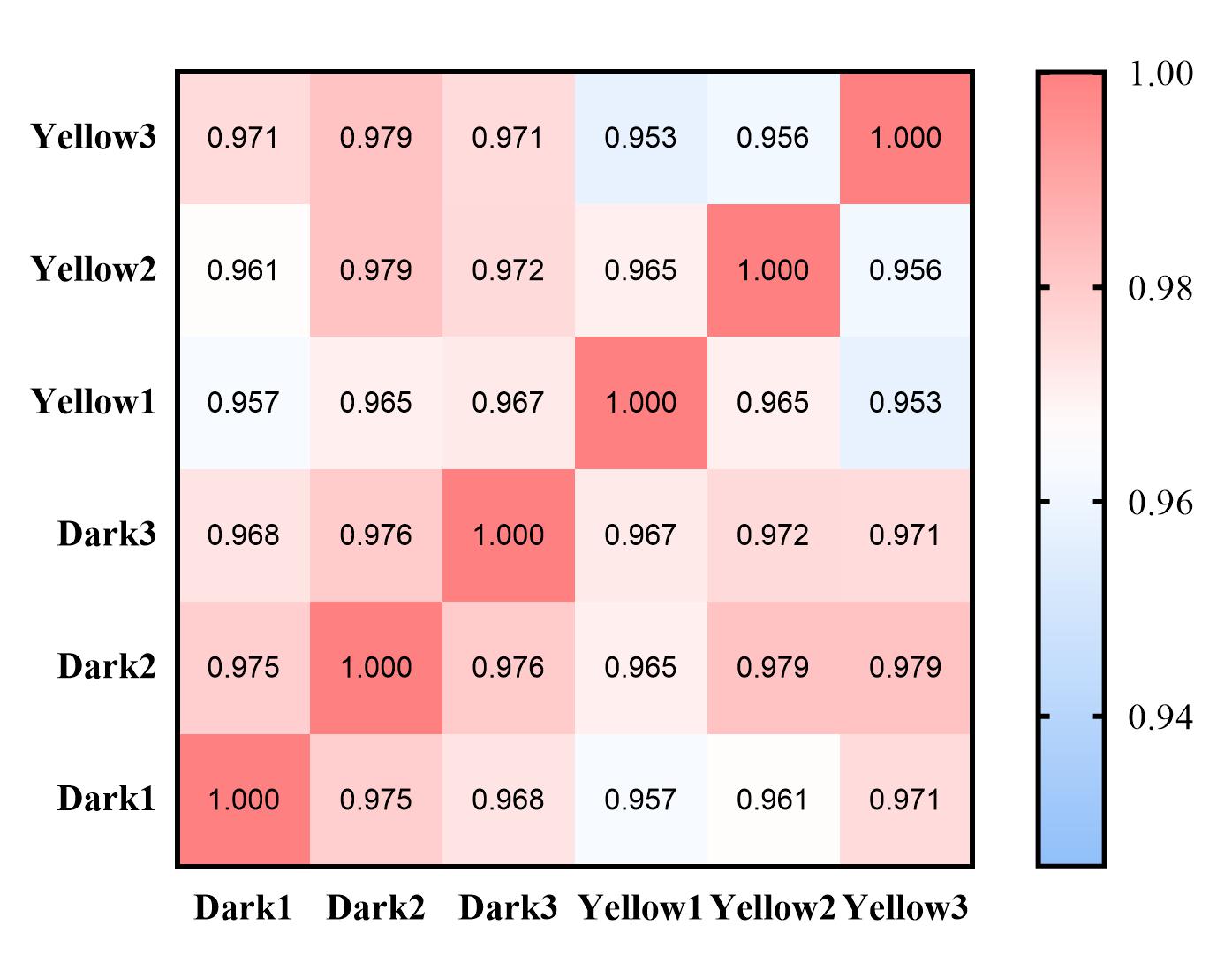


**Figure S2.** Pearson correlation between the transcriptomic data derived from samples cultured under yellow light and darkness. Each treatment was triplicated.


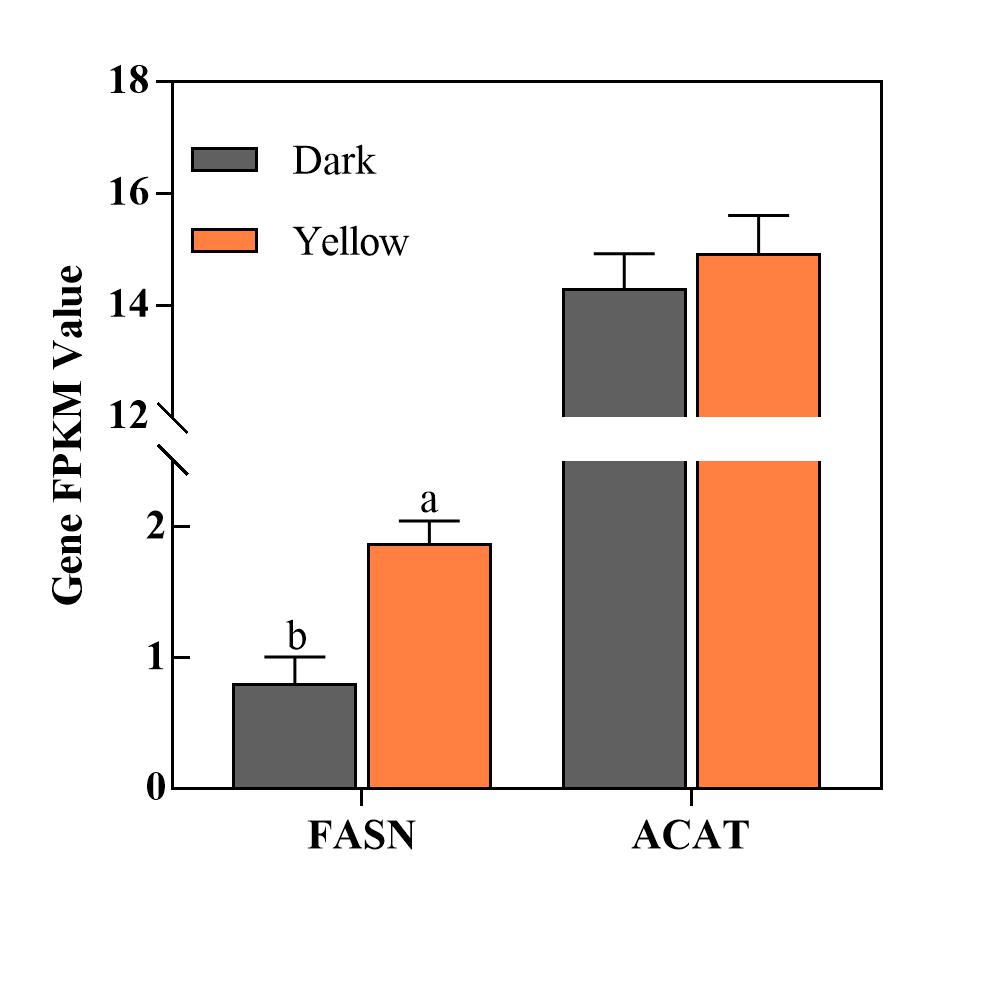


**Figure S3.** Relative FPKM value of fatty acid metabolic pathway related- genes in samples cultured under dark and yellow light. Values (mean±SD, n=3) sharing a common superscript are not significantly different (*P*>0.05).


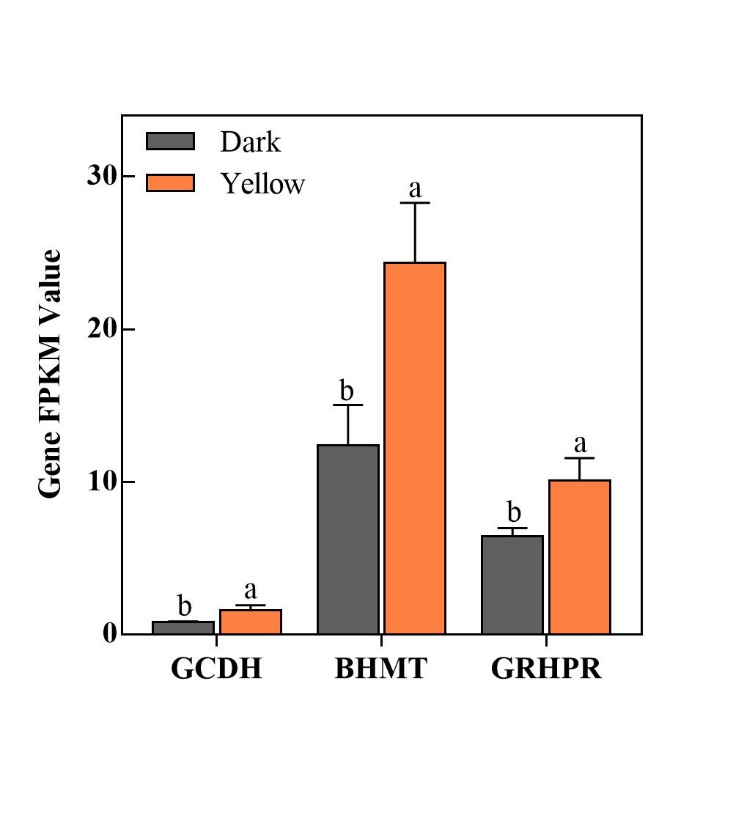


**Figure S4.** Relative FPKM value of amino acid metabolic pathway related- genes in samples cultured under dark and yellow light. Values (mean±SD, n=3) sharing a common superscript are not significantly different (*P*>0.05).


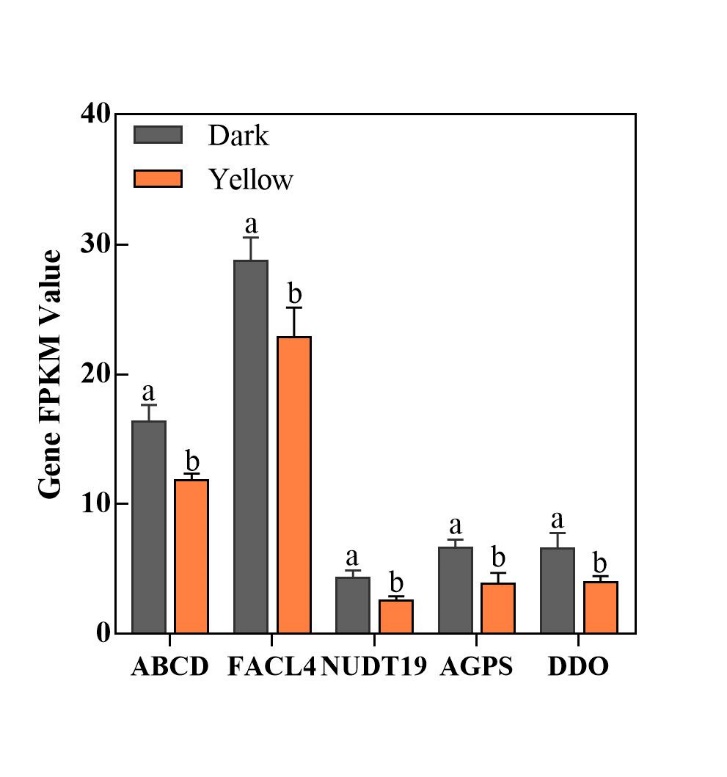


**Figure S5.** Relative FPKM value of peroxisome pathway related- genes in samples cultured under dark and yellow light. Values (mean±SD, n=3) sharing a common superscript are not significantly different (*P*>0.05).


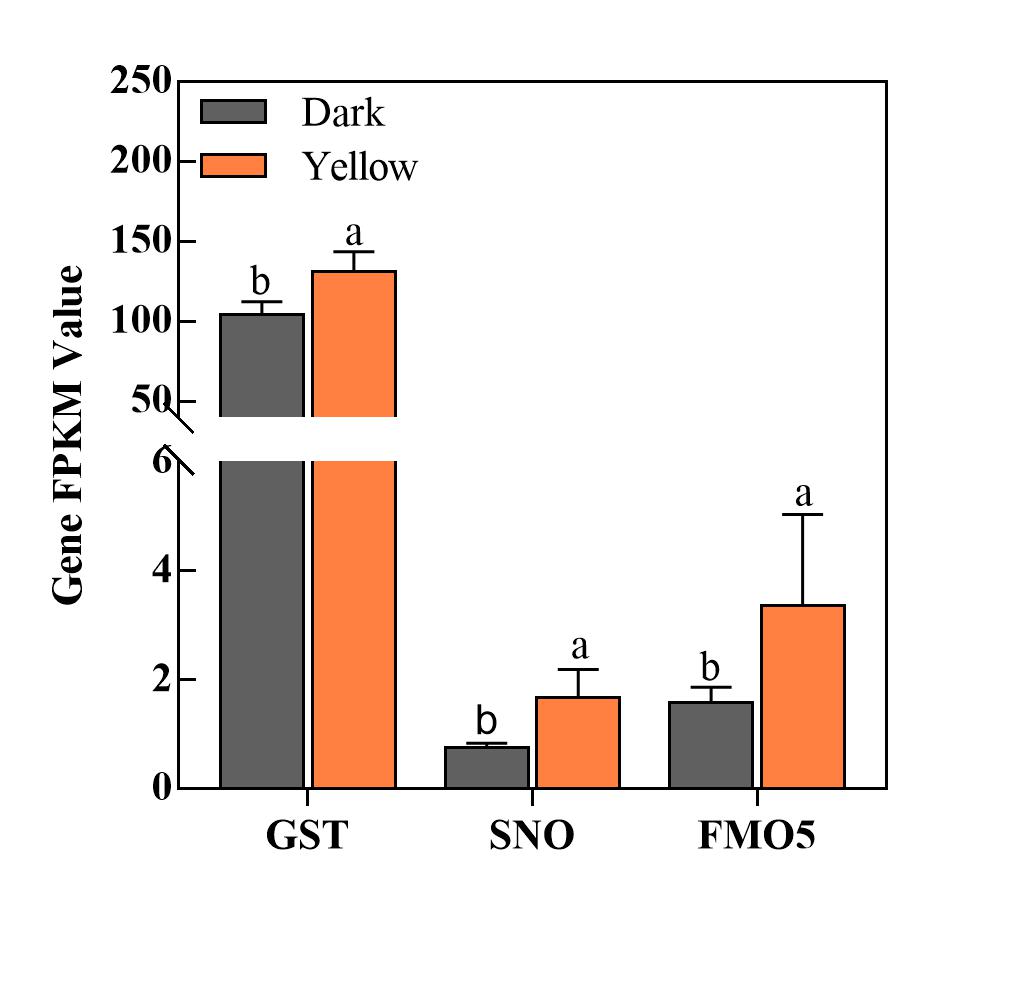


**Figure S6.** Relative FPKM value of Cytochrome P450 pathway related- genes in samples cultured under dark and yellow light. Values (mean±SD, n=3) sharing a common superscript are not significantly different (*P*>0.05).


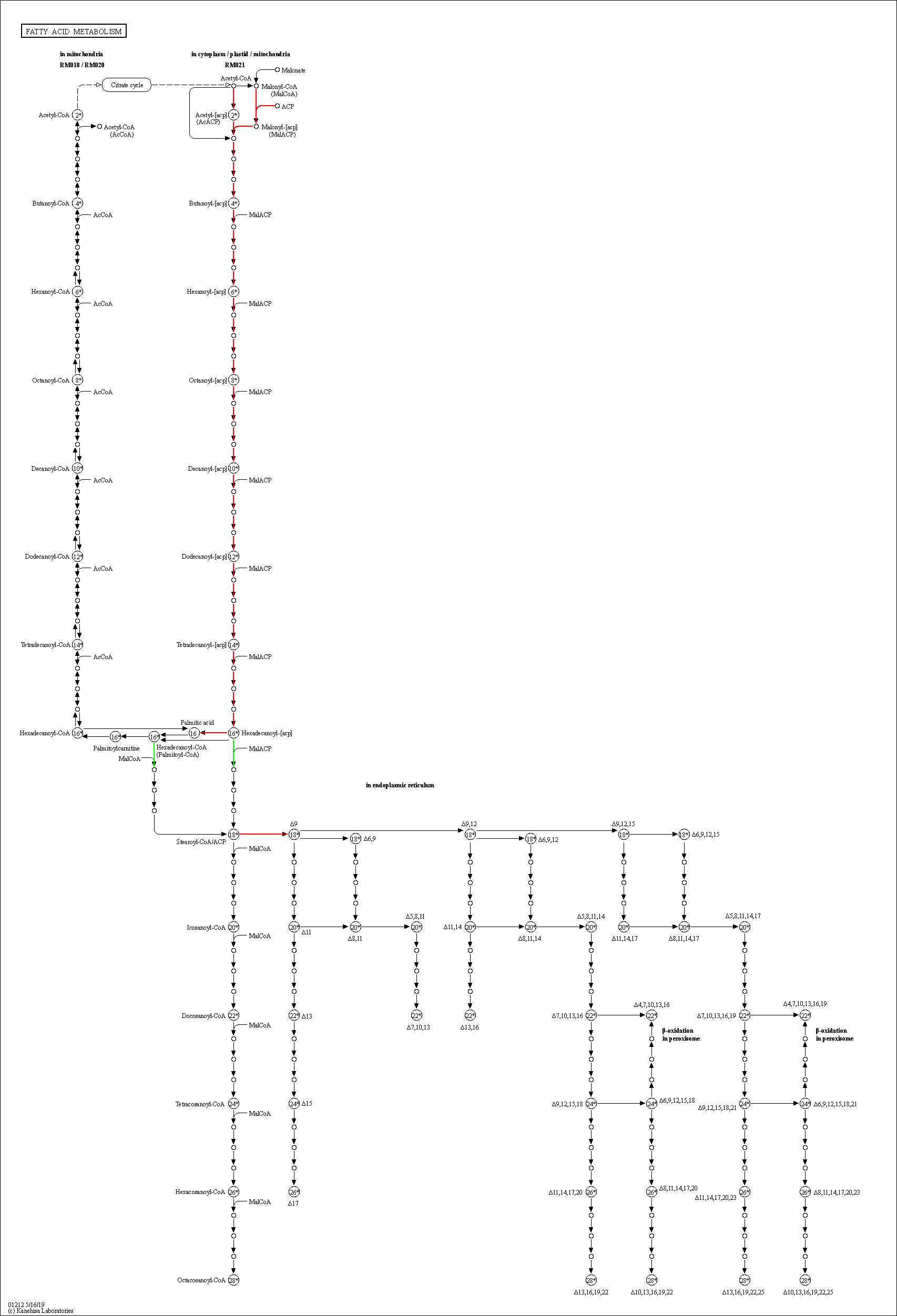


**Figure S7.** Comparison of expression patterns of genes in Fatty acid metabolism pathway of *S. constricta* cultured under dark and yellow light. Upregulated genes are indicated by red arrows, while downregulated genes are denoted in green.


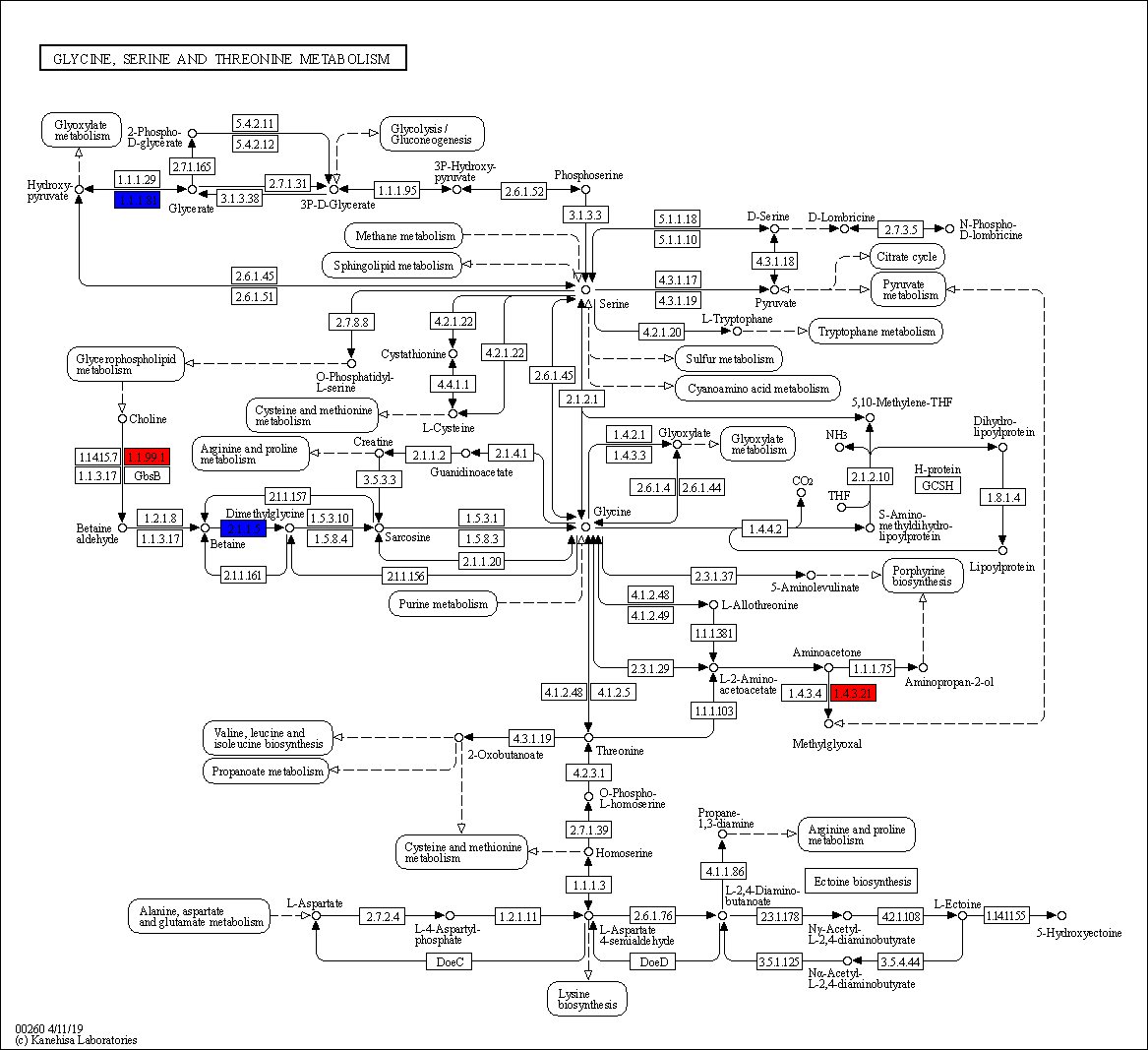


**Figure S8.** Comparison of expression patterns of genes in Glycine, serine and threonine metabolism of *S. constricta* cultured under dark and yellow light. Upregulated genes are indicated by red colors, while downregulated genes are denoted in blue.


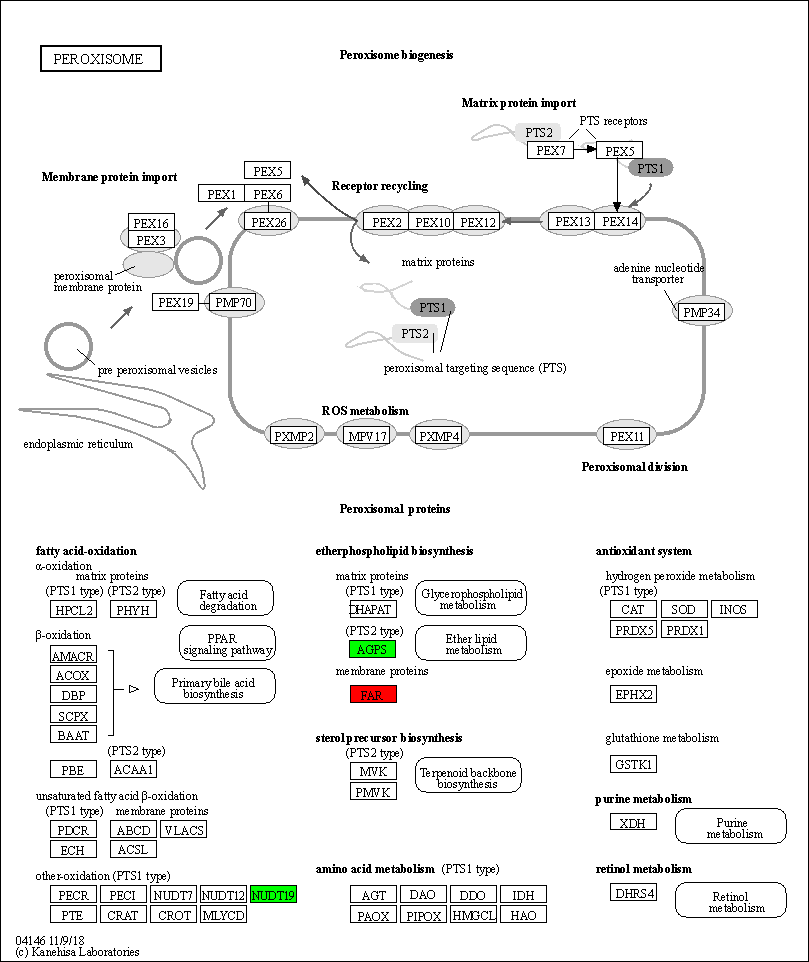


**Figure S9.** Comparison of expression patterns of genes in peroxisome pathway of *S. constricta* cultured under dark and yellow light. Upregulated genes are indicated by red colors, while downregulated genes are denoted in green.


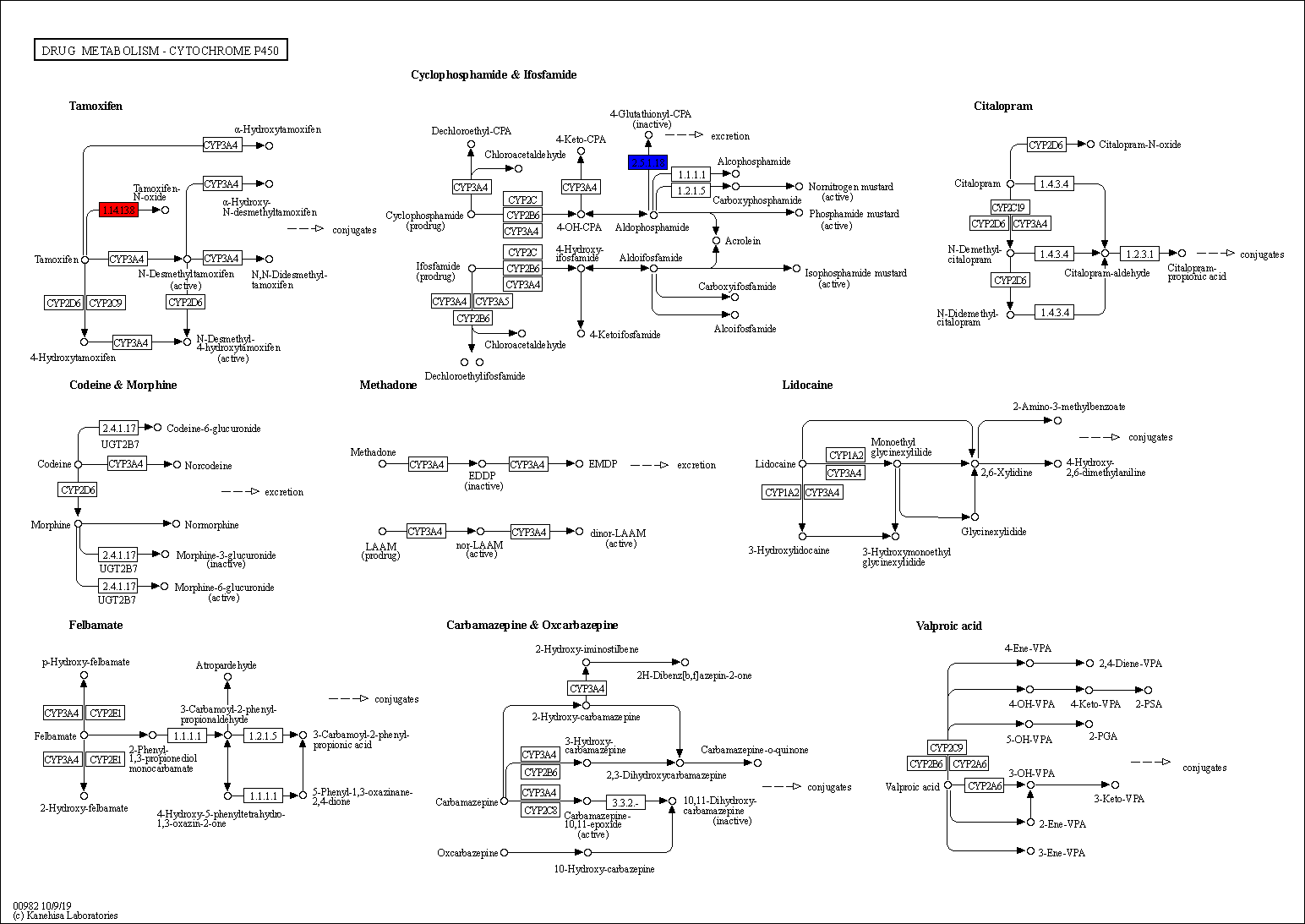


**Figure S10.** Comparison of expression patterns of genes in cytochrome P450 pathway of *S. constricta* cultured under dark and yellow light. Upregulated genes are indicated by red colors, while downregulated genes are denoted in blue.

**Supplementary Tables**

**Table S1** Fatty acid compositions (%) in *S. constricta* cultured under different light spectra.

| Fatty acid | Proportion at different light spectrum (%) | | | | | | | |
| --- | --- | --- | --- | --- | --- | --- | --- | --- |
|  | Dark | White | Violet | Blue | Cyan | Green | Yellow | Red |
| C14:0 | 1.28±0.08^b^ | 1.27±0.25^b^ | 1.68±0.02^a^ | 1.49±0.05^ab^ | 1.18±0.30^c^ | 1.12±0.04^c^ | 1.52±0.12^a^ | 1.33±0.11^b^ |
| C16:0 | 21.85±0.04^ab^ | 20.15±0.46^b^ | 24.62±0.15^a^ | 19.99±0.23^c^ | 21.73±0.81^ab^ | 20.42±0.09^b^ | 17.98±1.33^c^ | 20.86±0.67^b^ |
| C16:1(n-7) | 3.37±0.05^d^ | 4.24±0.05^a^ | 3.59±0.20^c^ | 4.25±0.11^ab^ | 4.05±0.05^b^ | 3.28±0.05^d^ | 4.22±0.05^ab^ | 3.59±0.14^c^ |
| C16:2(n-4) | 0.87±0.00^d^ | 1.18±0.09^c^ | 0.93±0.03^d^ | 0.99±0.03^d^ | 1.36±0.08^b^ | 0.93±0.05^d^ | 2.30±0.03^a^ | 0.94±0.05^d^ |
| C17:0 | 0.24±0.01^bc^ | 0.22±0.01^c^ | - | 0.30±0.01^a^ | 0.24±0.02^bc^ | 0.25±0.01^bc^ | 0.22±0.01^c^ | 0.27±0.03^ab^ |
| C17:3(n-4) | 1.20±0.07^d^ | 1.57±0.01^c^ | - | 1.84±0.08^ab^ | 1.51±0.03^c^ | 1.90±0.05^a^ | 1.81±0.02^ab^ | 1.72±0.06^b^ |
| C18:0 | 13.93±1.01^bc^ | 14.11±0.20^bc^ | 18.50±0.36^a^ | 14.68±0.44^bc^ | 14.96±0.74^bc^ | 15.53±0.52^bc^ | 12.27±0.41^c^ | 16.34±1.73^ab^ |
| C18:1(n-9) | 6.42±0.08^b^ | 5.60±0.06^d^ | 5.30±0.03^e^ | 6.68±0.06^a^ | 6.17±0.19^c^ | 5.91±0.12^d^ | 6.52±0.11^ab^ | 6.44±0.12^b^ |
| C18:1(n-7) | 6.25±0.08^a^ | 5.89±0.04^b^ | 5.94±0.01^a^ | 5.91±0.03^a^ | 6.30±0.28^a^ | 6.09±0.26^a^ | 6.31±0.06^a^ | 6.25±0.17^a^ |
| C18:2(n-6) | 0.62±0.04^abc^ | 0.66±0.01^a^ | 0.62±0.03^abc^ | 0.55±0.01^d^ | 0.58±0.01^cd^ | 0.60±0.02^bc^ | 0.60±0.01^bc^ | 0.64±0.02^ab^ |
| C18:3(n-6) | 1.10±0.03^bc^ | 1.05±0.02^bc^ | - | 1.18±0.02^b^ | 1.04±0.01^c^ | - | 1.09±0.05^bc^ | 1.26±0.12^a^ |
| C18:3(n-3) | 14.93±0.25^a^ | 14.62±0.46^a^ | 13.37±0.18^b^ | 14.16±0.33^b^ | 13.23±0.21^b^ | 15.33±0.06^a^ | 15.39±0.41^a^ | 13.53±0.27^b^ |
| C18:4(n-3) | 10.27±0.57^bc^ | 10.80±0.31^a^ | 9.69±0.18^c^ | 10.21±0.11^bc^ | 9.84±0.38^c^ | 9.99±0.13^c^ | 10.73±0.22^a^ | 8.35±0.33^d^ |
| C20:0 | 0.25±0.01^bc^ | 0.25±0.02^bc^ | 0.31±0.01^a^ | 0.28±0.03^b^ | 0.25±0.01^bc^ | 0.24±0.01^d^ | 0.19±0.01^d^ | 0.25±0.01^bc^ |
| C20:1(n-9) | 0.60±0.04^a^ | 0.61±0.09^a^ | 0.60±0.01^a^ | 0.60±0.01^a^ | 0.58±0.02^a^ | 0.59±0.06^a^ | 0.54±0.03^a^ | 0.58±0.06^a^ |
| C20:1(n-7) | 4.52±0.07^e^ | 5.04±0.05^a^ | 4.63±0.01^cd^ | 4.62±0.02^e^ | 4.82±0.05^bc^ | 5.23±0.01^a^ | 4.95±0.17^ab^ | 4.77±0.11^c^ |
| C20:2(n-7) | 0.42±0.02^ab^ | 0.45±0.01^a^ | 0.46±0.00^a^ | 0.48±0.01^a^ | 0.45±0.02^a^ | 0.44±0.01^ab^ | 0.45±0.01^a^ | 0.45±0.02^a^ |
| 20:4(n-6) | 0.56±0.03^bc^ | 0.49±0.01^c^ | 0.45±0.00^d^ | 0.56±0.01^bc^ | 0.42±0.00^d^ | 0.46±0.01^d^ | 0.60±0.01^ab^ | 0.71±0.02^a^ |
| 20:3(n-6) | 0.23±0.01^a^ | 0.24±0.00^a^ | 0.23±0.00^a^ | 0.20±0.00^b^ | 0.26±0.00^a^ | 0.26±0.00^a^ | 0.24±0.00^a^ | 0.25±0.01^a^ |
| 20:5(n-3) | 1.17±0.06^a^ | 1.12±0.09^ab^ | 0.90±0.01^c^ | 1.02±0.01^c^ | 1.15±0.12^ab^ | 1.07±0.18^bc^ | 1.17±0.02^a^ | 1.15±0.08^ab^ |
| 22:2 (5,13) | 0.59±0.01^b^ | 0.58±0.03^b^ | 0.52±0.00^c^ | 0.59±0.00^b^ | 0.61±0.00^ab^ | 0.60±0.01^b^ | 0.63±0.01^a^ | 0.59±0.02^b^ |
| 22:4(n-6) | 2.96±0.03^cd^ | 3.02±0.06^bc^ | 2.57±0.00^e^ | 2.95±0.03^d^ | 2.91±0.08^cd^ | 2.98±0.10^cd^ | 3.38±0.07^a^ | 3.16±0.12^b^ |
| 22:5(n-6) | 0.29±0.05^b^ | 0.37±0.00^a^ | - | 0.29±0.00^b^ | 0.26±0.01^c^ | 0.26±0.00^c^ | 0.32±0.00^a^ | 0.25±0.01^c^ |
| 22:6(n-3) | 5.93±0.03^c^ | 6.35±0.08^a^ | 5.92±0.02^c^ | 6.07±0.02^c^ | 5.97±0.09^c^ | 6.40±0.01^ab^ | 6.47±0.09^a^ | 6.20±0.21^b^ |
| SFA | 37.57±1.09^b^ | 36.02±0.52^b^ | 45.13±0.51^a^ | 36.76±0.57^b^ | 38.39±1.23^b^ | 37.58±0.40^b^ | 32.2±0.93^b^ | 39.07±1.22^b^ |
| MUFA | 21.18±0.27^bc^ | 21.4±0.11^bc^ | 20.08±0.21^c^ | 22.08±0.21^b^ | 21.94±0.31^b^ | 21.12±0.36^bc^ | 22.56±0.19^a^ | 21.65±0.52^bc^ |
| PUFA | 41.23±0.83^bc^ | 42.56±0.53^b^ | 34.77±0.39^c^ | 41.14±0.46^cd^ | 39.66±0.98^cd^ | 41.28±0.13^b^ | 45.23±0.80^a^ | 39.26±0.71^c^ |

Note: SFA: saturated fatty acid; MUFA: monounsaturated fatty acid; PUFA: polyunsaturated fatty acid; TFA: total fatty acid; “n.d.”: not detected. Date is expressed as mean ± SD (n = 3). Values in the same row sharing the same letter were not significantly different (*P* > 0.05)

**Table S2.** Amino acid compositions (%) in *S. constricta* cultured under different light spectra.

| Amino acid | Proportion at different light spectra (%) | | | | | | | |
| --- | --- | --- | --- | --- | --- | --- | --- | --- |
|  | Dark | White | Violet | Blue | Cyan | Green | Yellow | Red |
| Thr^1^ | 4.60±0.03^a^ | 4.36±0.16^ab^ | 4.47±0.05^ab^ | 4.37±0.10^ab^ | 4.21±0.2^bc^ | 4.04±0.05^c^ | 4.55±0.02^a^ | 4.28±0.10^abc^ |
| Val^1^ | 4.37±0.02^a^ | 4.19±0.12^bc^ | 4.35±0.00^ab^ | 4.23±0.09^ab^ | 4.06±0.09^cd^ | 4.02±0.10^d^ | 4.38±0.03^a^ | 4.19±0.13^bc^ |
| Ile^1^ | 4.60±0.04^a^ | 4.40±0.13^bcd^ | 4.57±0.00^ab^ | 4.43±0.06^abc^ | 4.24±0.07^de^ | 4.22±0.12^e^ | 4.58±0.01^a^ | 4.39±0.16^cde^ |
| Leu^1,3^ | 9.37±0.05^a^ | 9.14±0.24^ab^ | 9.35±0.06^a^ | 9.08±0.03^b^ | 8.80±0.03^cd^ | 8.66±0.09^d^ | 9.37±0.04^a^ | 8.96±0.30^bc^ |
| Met^1,3^ | 2.44±0.02^a^ | 2.38±0.03^a^ | 2.39±0.01^a^ | 2.40±0.06^a^ | 2.25±0.1^bc^ | 2.16±0.10^c^ | 2.45±0.04^a^ | 2.36±0.07^ab^ |
| Phe^1,3^ | 3.17±0.00^a^ | 3.07±0.08^abc^ | 3.15±0.00^ab^ | 3.08±0.09^abc^ | 3.01±0.10^cd^ | 2.93±0.04^d^ | 3.16±0.00^a^ | 3.02±0.08^bcd^ |
| Lys^1,3^ | 7.31±0.04^a^ | 7.20±0.20^ab^ | 7.33±0.02^a^ | 7.10±0.08^ab^ | 7.02±0.14^bc^ | 6.83±0.07^c^ | 7.33±0.02^a^ | 7.03±0.21^bc^ |
| Ser^2^ | 4.55±0.05^a^ | 4.55±0.10^a^ | 4.61±0.00^a^ | 4.22±0.11^b^ | 4.27±0.22^b^ | 4.13±0.00^b^ | 4.57±0.03^a^ | 4.27±0.14^b^ |
| Pro^2^ | 1.12±0.00^a^ | 1.11±0.03^a^ | 1.12±0.00^a^ | 1.09±0.04^ab^ | 1.09±0.04^ab^ | 1.04±0.00^b^ | 1.14±0.00^a^ | 1.10±0.03^a^ |
| Ala^2^ | 17.43±0.13^a^ | 17.45±0.05^a^ | 17.24±1.20^a^ | 16.9±0.32^a^ | 16.29±1.07^ab^ | 15.68±0.27^b^ | 16.28±0.24^ab^ | 16.65±0.47^ab^ |
| Glu^2,3^ | 13.55±0.04^a^ | 13.28±0.35^ab^ | 13.61±0.10^a^ | 13.27±0.16^ab^ | 12.76±0.06^c^ | 12.62±0.12^c^ | 13.59±0.12^a^ | 12.87±0.58^bc^ |
| Gly^2,3^ | 5.38±0.02^a^ | 5.05±0.21^a^ | 5.44±0.52^a^ | 5.19±0.39^a^ | 5.12±0.78^a^ | 5.48±0.01^a^ | 5.84±0.12^a^ | 5.10±0.57^a^ |
| Asp^2,3^ | 9.28±0.01^a^ | 9.03±0.21^ab^ | 9.23±0.06^a^ | 9.03±0.16^ab^ | 8.60±0.19^cd^ | 8.54±0.08^d^ | 9.31±0.00^a^ | 8.87±0.28^bc^ |
| Arg^3^ | 7.37±0.00^ab^ | 7.40±0.41^ab^ | 7.31±0.14^ab^ | 7.49±0.41^ab^ | 7.42±0.52^ab^ | 7.04±0.15^b^ | 7.79±0.33^a^ | 7.33±0.19^ab^ |
| Tyr^3^ | 2.71±0.02^ab^ | 2.66±0.11^ab^ | 2.66±0.04^ab^ | 2.69±0.10^ab^ | 2.68±0.21^ab^ | 2.49±0.05^b^ | 2.77±0.06^a^ | 2.63±0.12^ab^ |
| Cys | 0.69±0.00^a^ | 0.69±0.03^a^ | 0.73±0.00^a^ | 0.75±0.08^a^ | 0.71±0.07^a^ | 0.60±0.02^b^ | 0.76±0.02^a^ | 0.71±0.02^a^ |
| His | 2.01±0.00^ab^ | 1.95±0.04^ab^ | 2.00±0.01^ab^ | 1.97±0.05^ab^ | 1.88±0.03^c^ | 1.87±0.02^c^ | 2.01±0.00^a^ | 1.94±0.04^b^ |
| TEAA | 35.88±0.23^a^ | 34.78±0.94^bc^ | 35.63±0.17^ab^ | 34.71±0.34^bc^ | 33.63±0.24^de^ | 32.90±0.60^e^ | 35.85±0.04^a^ | 34.26±0.99^cd^ |
| TFAA | 51.33±0.16^a^ | 50.50±0.56^ab^ | 51.28±0.50^a^ | 49.73±0.91^bc^ | 48.15±0.75^de^ | 47.51±0.07^e^ | 50.75±0.02^ab^ | 48.88±1.48^cd^ |
| TMAA | 60.61±0.24^ab^ | 59.26±1.59^abc^ | 60.52±1.00^ab^ | 59.37±1.21^abc^ | 57.70±1.53^cd^ | 56.80±0.73^d^ | 61.66±0.13^a^ | 58.21±2.26^bcd^ |

Note: ^1^Essentital amino acid; ^2^Flavor amino acid; ^3^Medical amino acid; Thr: threonine; Val: valine; Ile: isoleucine; Leu: leucine; Met: methionine; Phe: phenylalanine; Lys: lysine; Ser: serine; Pro: proline; Ala: alanine; Glu: glutamic acid; Gly: glycine; Asp: aspartic acid; Arg: arginine; Tyr: tyrosine; Cys: cysteine; His: histidine. TEAA: total essential amino acid; TFAA: total flavor amino acid; TMAA: total medicinal amino acid; TAA: total amino acid. Date is expressed as mean ± SD (n = 3). Values in the same row sharing the same letter were not significantly different (*P* > 0.05).

**Table S3.** Summary of sequencing and mapping of juvenile *S. constricta* transcriptome.

| Sample | | Total reads | | Clean bases | Q20 (%) | Q30 (%) | GC (%) | Total mapped |
| --- | --- | --- | --- | --- | --- | --- | --- | --- |
| Dark1 | 55987650 | | 8,377,383,492 | | 99.10 | 94.43 | 40.30 | 40,963,307 (73.16%) |
| Dark2 | 60060642 | | 8,986,521,580 | | 99.18 | 94.93 | 41.24 | 44,771,536 (74.54%) |
| Dark3 | 45472540 | | 6,805,546,542 | | 99.13 | 94.62 | 41.82 | 34,289,141 (75.41%) |
| Yellow1 | 51784152 | | 7,747,647,632 | | 99.10 | 94.44 | 40.52 | 37,946,116 (73.28%) |
| Yellow 2 | 55288572 | | 8,274,491,734 | | 99.07 | 94.27 | 41.84 | 41,791,569 (75.59%) |
| Yellow 3 | 57006354 | | 8,529,596,530 | | 99.15 | 94.74 | 41.21 | 42,008,856 (73.69%) |
